# Radar Tracking and Motion-Sensitive Cameras on Flowers Reveal the Development of Pollinator Multi-Destination Routes over Large Spatial Scales

**DOI:** 10.1371/journal.pbio.1001392

**Published:** 2012-09-20

**Authors:** Mathieu Lihoreau, Nigel E. Raine, Andrew M. Reynolds, Ralph J. Stelzer, Ka S. Lim, Alan D. Smith, Juliet L. Osborne, Lars Chittka

**Affiliations:** 1 Biological and Experimental Psychology Group, School of Biological and Chemical Sciences, Queen Mary University of London, London, United Kingdom; 2 Rothamsted Research, Harpenden, Hertfordshire, United Kingdom; University of Sussex, United Kingdom

## Abstract

Central place foragers, such as pollinating bees, typically develop circuits (traplines) to visit multiple foraging sites in a manner that minimizes overall travel distance. Despite being taxonomically widespread, these routing behaviours remain poorly understood due to the difficulty of tracking the foraging history of animals in the wild. Here we examine how bumblebees (*Bombus terrestris*) develop and optimise traplines over large spatial scales by setting up an array of five artificial flowers arranged in a regular pentagon (50 m side length) and fitted with motion-sensitive video cameras to determine the sequence of visitation. Stable traplines that linked together all the flowers in an optimal sequence were typically established after a bee made 26 foraging bouts, during which time only about 20 of the 120 possible routes were tried. Radar tracking of selected flights revealed a dramatic decrease by 80% (*ca.* 1500 m) of the total travel distance between the first and the last foraging bout. When a flower was removed and replaced by a more distant one, bees engaged in localised search flights, a strategy that can facilitate the discovery of a new flower and its integration into a novel optimal trapline. Based on these observations, we developed and tested an iterative improvement heuristic to capture how bees could learn and refine their routes each time a shorter route is found. Our findings suggest that complex dynamic routing problems can be solved by small-brained animals using simple learning heuristics, without the need for a cognitive map.

## Abstract 2

Automated tracking of bumblebees and computer simulations reveal how bees locate a series of flowers and optimize their routes to visit them all.

## Author Summary

Many food resources, such as flowers refilling with nectar or fruits ripening on a tree, replenish over time, so animals that depend on them need to develop strategies to reduce the energy they use during foraging. Here we placed five artificial flowers in a field and set out to examine how bumblebees optimize their foraging routes between distant locations. We tracked the flight paths of individual bees with harmonic radar and recorded all their visits to flowers with motion-sensitive video cameras. This dataset allowed us to study how bees gradually discover flowers, learn their exact position in the landscape, and then find the shortest route to collect nectar from each flower in turn. Using computer simulations, we show that the level of optimisation performance shown by bees can be replicated by a simple learning algorithm that could be implemented in a bee brain. We postulate that this mechanism allows bumblebees to optimise their foraging routes in more complex natural conditions, where the number and productivity of flowers vary.

## Introduction

Animals moving in familiar environments often follow habitual routes to navigate between important locations, such as the nest and feeding sites. Most knowledge on route following behaviours has been deduced from the stereotyped paths insects [1]–[4] and birds [5] develop when travelling between home and a single other site. In contrast, very little is known about the routing decisions made by animals that must visit multiple sites before returning home. These routing challenges are common in central place foraging nectarivores and frugivores, which typically exploit familiar food resources that replenish over time. Many of these animals develop stable foraging circuits (traplines) between distant food patches [6]–[10] and must sometimes cover several kilometres to fill their crop [11].

Developing an efficient route to reduce the travelling costs between multiple foraging locations is an optimisation task analogous to the well-known travelling salesman problem (finding the shortest route to visit a set of locations once and return to the origin) [12]. The most direct approach to solve this mathematical problem is to compare all the possible routes, which often requires extensive computational power as the number of routes increases factorially with the number of locations to be visited (e.g., 5! = 120 possible routes in a problem with only 5 locations). For animals, this problem is of a different nature as they cannot plan a route in advance, using a geographic map, but must gradually acquire information about the locations and the paths linking them. Therefore many animals [13]–[16], including humans [17],[18], navigating between multiple locations are thought to find efficient routes using heuristic strategies, such as linking nearest unvisited sites or planning a few steps ahead.

Recent laboratory studies have shown that bumblebees foraging in simple arrangements of artificial flowers in indoor flight cages develop near optimal traplines after extensive exploration, based on learning and spatial memories [15],[19]–[21]. However, whether similar strategies are observed at larger spatial scales, when animals must search to localise distant feeding sites and when the costs of travelling suboptimal routes are magnified, remains largely unexplored. In addition, over the smaller spatial scales at which bees were previously tested, nearby flowers were typically visible from other flowers, which is often not the case over natural foraging scales in the field. Obtaining data about the ontogeny of traplines in the wild is challenging, since it requires the observer to have information about the spatial location of all available food patches, the complete foraging history of the animals, and their movements with sufficient accuracy to retrace their routes.

Here, taking advantage of the possibility to train bumblebees (*Bombus terrestris*) to forage on artificial flowers in the field [22], to track their complete flight paths with harmonic radar [23],[24], and to record all their flower visits with motion-sensitive cameras, we investigate the acquisition of long-distance traplines by animals with known foraging experience. We describe how bees develop stable routes between five feeding locations by combining exploration, learning, and sequential optimization. We then compare bees' optimization performances to those of simple heuristic algorithms and develop a novel iterative improvement heuristic replicating the observed dynamics of route acquisition.

## Results

### Trapline Development between Five Flowers Arranged in a Regular Pentagon

**Figure 1:**
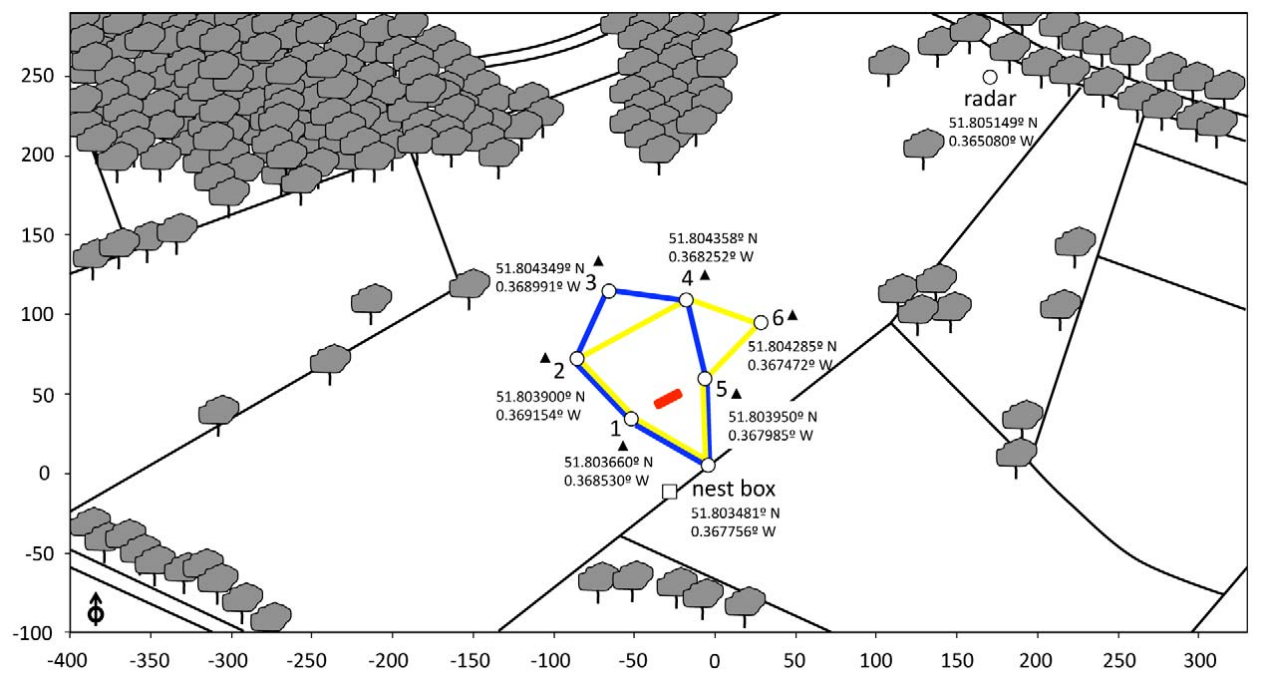
Aerial view of the experimental field. The area was structured both by landmarks providing global references (edges between different types of cut grass, lines of trees) and by local features (isolated trees). Naïve bees were pre-trained to forage on the five artificial flowers positioned in a linear array midway between location 1 and 5 (red line). In the first phase of the experiment, bees were observed foraging on the five flowers positioned in locations 1–5. The shortest possible route to visit all five flowers once and return to the nest box was 311.8 m long (blue line). In the second phase of the experiment, the flower at location 3 was removed and a new flower was established at location 6 (50 m from both location 4 and location 5). The shortest possible route in the modified array was 342.6 m (yellow line). In both spatial arrangements, the minimum distance between nearest neighbour flowers was 50 m. Open white arrow (bottom left) indicates north. White square indicates the location of the anemometer station. Black triangles represent the locations of the small generators used to power the motion detection equipment at each feeding station. GPS data (WGS 84) were recorded on an iPad (Apple, Cupertino, CA). Satellite image from Rothamsted estate, Hertfordshire, UK (http://maps.google.com). Scale is in metres.

Our first aim was to establish whether bees develop repeatable foraging circuits between stable feeding locations. We pre-trained naïve bees to collect sucrose solution rewards from a patch of five artificial flowers (Figure S1) in the middle of the experimental field (Figure 1). After a day of pre-training, bees of known foraging experience were tested individually with the five flowers arranged in regular pentagon (50 m side length). Each flower provided a sucrose reward equivalent to one-fifth of the bee's crop capacity and was refilled after each foraging bout. We tested seven bees for seven consecutive hours each on a different day. All visits to the flowers were video recorded with motion-activated webcams at each feeding station (Video S1). The first flight of an inexperienced forager and the final flight paths of five experienced foragers were recorded with harmonic radar (Videos S2, S3, S4, S5, S6, S7, S8, S9, S10, S11, S12, S13).

**Figure 2:**
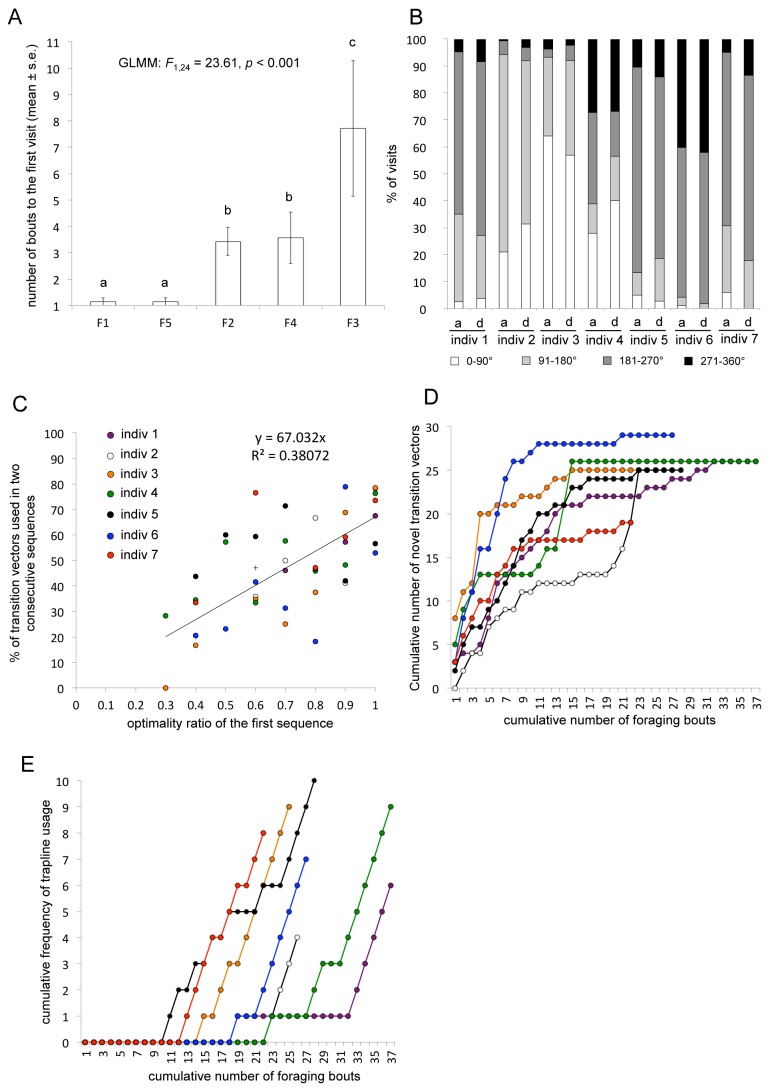
Trapline development in the initial pentagonal array of flowers. (A) The average number of foraging bouts (mean ± s.e.m., *n* = 7 bees) before a bee made its first visit to each flower (F1–F5). Letters above columns indicate significant differences (GLMM, effect of flower on the number of bouts, *t* test: *p*<0.05). (B) Proportion of arrivals (a) and departures (d) made by each bee in the four quadrants (90° sectors) of the landing platform on all flower visits. (C) Proportion of transition vectors (either nest-flower, flower-flower, or flower-nest) repeated in two successive bouts in relation to the optimality ratio (straight line length of the observed visitation sequence divided by the straight line length of the shortest possible sequence to visit the same number of flowers) of the first bout. (D) Cumulative frequency of different transition vectors experienced in relation to the cumulative number of foraging bouts completed. Each bee used on average 25±1.13 different vectors (mean ± s.e.m., *n* = 7 bees) out of a total of 30 possible. (E) Cumulative frequency of trapline usage (the most common five-flower visitation sequence, excluding revisits, used by each bee) in relation to the cumulative number of foraging bouts completed. Traplines were first observed between bout 11 and 23, and became stabilized (used in at least three consecutive bouts at the end of training) in six bees between bouts 24 and 35. Labels 1–7 refer to the same individuals in all figures and tables.

Bees discovered flowers sequentially and had visited all five flowers at least once after an average of eight foraging bouts (here, and throughout the text, means are reported ± s.e.m.; 8.14±2.43 bouts, *n* = 7 bees; Figure 2A). The two flowers closest to the nest (F1 and F5) were located first, by all individuals. The flower furthest from the nest (F3) was found last by four bees, whereas it was the penultimate flower discovered by the other three. Individual bees consistently approached flowers from the same quadrant of the landing platform (Video S1), irrespective of the flower visited and of their experience (Generalized Linear Mixed Model (GLMM), effect of quadrant on the frequency of visits, *F*_3,1328_ = 90.23, *p*<0.001; effect of flower identity, *F*_4,1328_ = 1.82, *p* = 0.08; effect of the number of bouts completed, *F*_1,1328_ = 0.07, *p* = 0.791; all interactions, *p*>0.05). Frequency distributions of approaches in each quadrant were significantly different among bees (*χ* ^2^_18_ = 996, *p*<0.05; Figure 2B), indicating that each bee approached and landed on flowers from a different preferred angle. Furthermore, bees departed from the same quadrant as they arrived (and thus in opposite directions) in 71.41%±1.72% (*n* = 7 bees) of visits. The frequency of visits when arrivals and departures occurred in the same quadrant did not vary significantly in relation to flower location or to the foraging experience of bees (GLMM, effect of flower identity on the frequency of visits where arrival and departure occurred in the same quadrant, *F*_4,1328_ = 2.27, *p* = 0.065; effect of the number of bouts completed, *F*_1,1328_ = 0.46, *p* = 0.499; interaction, *F*_4,1328_ = 4.87, *p* = 0.222). We also found no significant difference in the frequency of these visits among bees (*χ* ^2^_6_ = 10.29, *p* = 0.113). Therefore, our data suggest that each bee acquired a directional preference in arrivals to and departure from flowers before the observations began, possibly during the pre-training phase when the bees became familiar with the flower design, and used their directional preference consistently for visiting flowers in all novel locations discovered.

As they gained experience, bees increased the number of different flowers visited per foraging bout (first five bouts, 2.29±0.35 flowers; last five bouts, 4.97±0.06 flowers; *n* = 7 bees; GLMM, effect of the number of bouts completed on the number of flowers visited, *F*_1,194_ = 149.62, *p*<0.001) and reduced the frequency of revisits to empty flowers (first five bouts, 2.83±0.58 revisits; last five bouts, 1.31±0.55 revisits; *n* = 7 bees; GLMM, effect of the number of bouts completed on the frequency of revisits, *F*_1,194_ = 6.50, *p* = 0.012). In every bout, a bee's probability to link the nest and a flower or to link two flowers together was determined by its experience. Thus, transition vectors between any two locations used in previous bouts were used more often in subsequent bouts than transitions vectors never previously experienced (GLMM, effect of the cumulative frequency of all possible transition vectors in previous bouts on their frequency of usage at each bout, *F*_1,5848_ = 1,209.5, *p*<0.001). Among the paths already used, the probability of repeating a transition vector in two successive foraging bouts increased significantly with the optimality ratio (straight line length of the observed visitation sequence divided by straight line length of the shortest possible sequence to visit the same number of flowers) of the first bout (GLMM, effect of optimality ratio of the first bout on the frequency of transition vectors repeated in the second bout, *F*_1,1069_ = 82.64, *p*<0.001; Figure 2C). In other words, transition vectors that generated short routes were likely to be used again in subsequent bouts, while transition vectors producing long routes were gradually abandoned, thus limiting the number of novel transitions over time (Figure 2D).

With increasing experience, the sequence in which flowers were visited became more similar over successive foraging bouts (similarity index—see Materials and Methods—between the first two bouts, 0.2±0.05; similarity index between the last two bouts, 0.89±0.07, *n* = 7 bees; GLMM, effect of the number of bouts completed on similarity index, *F*_1,187_ = 78.14, *p*<0.001), leading to a regular repeatable sequence, or “trapline”: the most common five-flower visitation sequence excluding revisits used by each individual bee (Figure 2E; Table S1). On average, the trapline was used in 27.13%±3.46% (*n* = 7 bees) of each bee's foraging bouts. It first appeared after 17.57±1.79 bouts (*n* = 7 bees) and was stabilized (repeated in at least three consecutive bouts at the end of training) in six bees after 30±0.8 bouts. Among the 120 possible sequences to visit all five flowers once and return to the nest, each bee selected one of the two shortest possible sequences as its trapline, either by visiting the flowers in a clockwise (sequence, 12345; *n* = 4 bees) or an anti-clockwise order (sequence, 54321; *n* = 3 bees).

**Figure 3:**
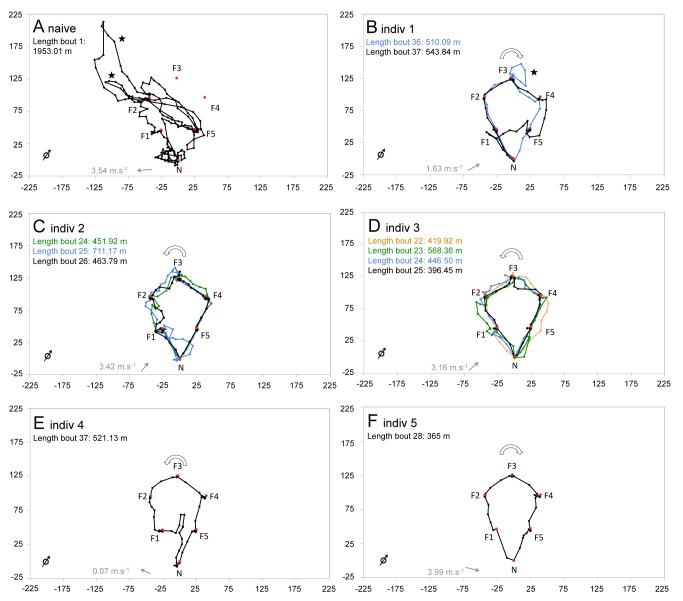
Flight paths in the initial pentagonal array of flowers. Black dots show the position of bees at 3 s intervals as recorded by the radar. Red dots indicate the locations of the artificial flowers (F1–F5) and the nest-box (N). (A) First flight of a naïve bee (for further tracks, see Figure S2). (B–F) Flight paths of experienced bees towards the end of training (flight paths for each bout are plotted in different colours, with the final flight in black). White arrows indicate the general directionality of the flower visitation sequence (clockwise or anticlockwise). Grey arrows indicate mean wind direction (degrees) and speed (m.s^−1^). The open black arrow (bottom left of each panel) indicates north. Stars indicate search loops between immediate revisits to flowers. Distances are in metres. Labels 1–5 refer to the same individuals in all figures and tables. Videos of all radar tracks are available (Videos S2, S3, S4, S5, S6, S7, S8, S9, S10, S11, S12, S13).

Radar tracks obtained from five experienced bees, near the end of the training phase, confirmed that the routes followed were highly repeatable and close to minimizing the overall travel distance (Figure 3B–F; Videos S3, S4, S5, S6, S7, S8, S9, S10, S11, S12, S13). Flight paths were composed of relatively straight segments linking either the nest and a flower or two flowers together. During each bee's final foraging bout, these flight segments were on average 26.09%±0.10% (*n* = 30 segments) longer than a straight line. Overall, the bees travelled 458.10±29.14 m (*n* = 5 bees), which is 146.92±29.14 m longer than the shortest possible path to visit the five flowers (311.8 m). This value contrasts sharply with the 1,953.01 m travelled by a naïve bee during its first foraging bout in the pentagonal array (Figure 3A; Video S2; for further tracks see Figure S2). Thus, over multiple bouts, bees effectively minimized their travel distances using a relatively direct path to visit all flowers once in an optimal order.

### Trapline Adjustment after Removal of a Flower and Introduction of a More Distant One

Our second aim was to investigate how experienced bees modify their trapline in response to changes in the spatial configuration of flowers. Immediately after radar-tracking the bees in the regular pentagonal array, we removed the flower located in the top corner (location 3) and established a new flower east of the initial pentagon (location 6). This new location was chosen to maximise the probability that search flights would be performed in the catchment area of the radar (Figure 1). We recorded the flight paths of three of the seven trained bees for eight consecutive foraging bouts (Videos S14, S15, S16, S17, S18, S19, S20, S21, S22, S23, S24, S25, S26, S27, S28, S29, S30, S31, S32, S33, S34, S35, S36, S37).

**Figure 4:**
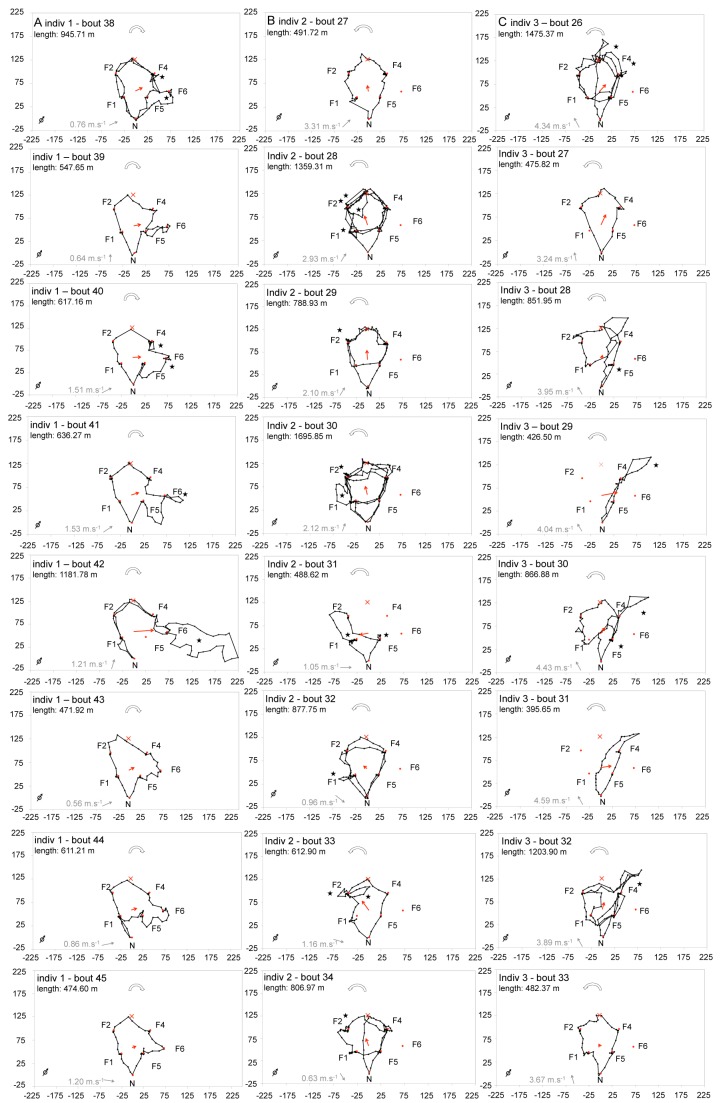
Flight paths in the modified pentagonal array of flowers. Red dots indicate the location of artificial flowers (F1, F2, F4, F5, F6) and the nest-box (N). One familiar flower (location 3) was removed and a new one was introduced (location 6). The red cross indicates the empty feeding location (artificial flower removed, formerly F3). Black dots show the position of the bees at 3 s intervals as recorded by radar. Movement patterns of the three bees were recorded during eight successive foraging bouts (one column per bee, flight paths shown in chronological order from top to bottom). Red arrows represent the mean flight vector of the flight path (see Materials and Methods). Stars indicate search loops between immediate revisits to flowers. White arrows indicate the general directionality of the flower visitation sequence (clockwise or anticlockwise). Grey arrows indicate mean wind direction (degrees) and speed (m.s^−1^). The open black arrow (bottom left of each panel) indicates north. Distances are in metres. Labels 1–3 refer to the same individuals in all figures and tables. Videos of all radar tracks are available (Videos S14, S15, S16, S17, S18, S19, S20, S21, S22, S23, S24, S25, S26, S27, S28, S29, S30, S31, S32, S33, S34, S35, S36, S37).

After the removal of the familiar flower, the bees increased their flight duration by around five times (last bout in initial array, 245.00±32.87 s; first bout in modified array, 1221.67±894.81 s; *n* = 3 bees), their travel distance more than doubled (last bout in initial array, 455.75±33.91 m; first bout in modified array, 970.93±284.24 m; *n* = 3 bees), and they once again started to revisit empty flowers (last bout in initial array, 0 revisits; first bout in modified array, 4.33±2.33 revisits; *n* = 3 bees). Bees continued to follow their trapline, visiting all four familiar flowers and the empty feeding location (location 3) in the same sequence as before the spatial arrangement was modified (Figure 4, Table S1). However, as bees could not fill their crop to capacity by visiting only four flowers, they repeated the entire circuit once, sometimes twice before returning to the nest, a stereotyped pattern observed in 33.33%±15.02% of all their foraging bouts (*n* = 8 bouts per bee).

At the same time, bees engaged in local searching manoeuvres, exploring new areas of the experimental field (Figure 4). Azimuthal directions of the mean flight vectors (sum of all vectors of the radar track, see Materials and Methods) indicate that bees did not investigate the entire field (Watson's test for circular uniformity, *p*<0.01 for every bee), but each one restricted their searching activity to a different sector (average angle for individual 1, 75.09±2.91°; individual 2, −32.17±11.40°; individual 3, 32.38±13.14°; *n* = 8 tracks per bee; ANOVA for circular data, *F*_2,23_ = 30.31, *p*<0.001). Sixteen out of 24 flight paths included loops of varying length (range, 5.10–509.26 m) between immediate revisits to the same flower (Figure 4). During these loops, the bees' ground speed was significantly slower than during other nest-flower or flower-flower flight segments (speed during loop, 1.90±0.28 m.s^−1^, *n* = 25 loops; speed during segment, 3.72±0.07 m.s^−1^, *n* = 173 segments; GLMM, effect of flight type on speed, *F*_1,197_ = 41.16, *p*<0.001). Slow flight loops were also frequent in the paths of the naïve bee (loop length, 171.60±95.47 m, *n* = 12 loops; speed during loop, 1.49±0.61 m.s^−1^; bouts 1–4 in Figure S2), and were observed only once in the paths of experienced bees in the initial spatial arrangement (bout 36 of individual 1 in Figure 3B). This difference in flight speed suggests that bees alternated between phases of exploitation characterized by relatively fast and straight flight segments and phases of exploration characterized by slow and localised flight loops. A similar pattern has been observed in displaced honeybees, which typically exhibit fast vector flights in the expected direction of a familiar location followed by slow search curves after finding that the target is not in its expected location [25].

One bee (individual 1) found the new flower location during its first foraging bout following the rearrangement of flowers (Figure 4A), integrated it into a new optimal sequence (sequence, 12465) during the third bout, and gradually stabilized this new sequence into a trapline. The other two bees (individuals 2 and 3) confined their searching activity in different azimuthal directions and never found the new flower during the eight foraging bouts (Figure 4B and 4C). Wind direction had no significant influence on the bees' searching direction (correlation coefficient for angular variables, *r* = −0.21 *p* = 0.307). Thus, after the removal of a familiar flower, bees increased their frequency of immediate revisits to flowers exhibiting slow loops. These localised search flights might facilitate the discovery of new flowers by allowing bees to learn the spatial characteristics of new sectors of their environment, while still exploiting familiar flowers along their established trapline.

### Trapline Optimisation by Iterative Improvement

Having established that bees develop optimal traplines without trying all possible solutions and start exploring again if some flowers are removed from and/or introduced to the array, we further examined bees' optimisation strategy by comparing the observed visitation sequences to sequences generated by simple optimisation heuristics.

First, we tested the “nearest neighbour” heuristic, in which a model bee chooses the nearest unvisited flower as its next move until all flowers have been visited. This heuristic has been suggested to explain the routing behaviour of some animals [13],[14],[17],[19], including bees [19], at small spatial scales. When applied to our experimental situation (five flowers arranged in a regular pentagon) the nearest neighbour heuristic predicts that bees should always move between neighbouring flowers along the edges of the pentagon. Although a large proportion of the bees' movements involved linking nearest neighbour flowers, especially in the early bouts when all flowers were not yet discovered (77% of all transitions between flowers, *n* = 50 bouts) and after the stabilization of an optimal trapline (100% of all transitions between flowers, *n* = 19), this unique rule of thumb is not sufficient to fully explain our data since bees were observed moving between non-nearest neighbour flowers in 52% of the bouts in which all five flowers were visited (*n* = 42 bouts; Table S1).

Second, we tested the “discovery order” heuristic in which a model bee visits flowers in the order it discovered them. This heuristic has been previously proposed for the establishment of long-distance traplines by bees [16]. However, we found it incompatible with our observations as none of the bees used the discovery order of the flowers as their trapline sequence (Table S1). There was no significant relationship between the discovery order of the flowers and the directionality (clockwise or anti-clockwise) of final traplines (GLMM, effect of discovery order of flowers on their order in the trapline, *F*_1,29_ = 0.04, *p* = 0.844). For each bee, the similarity index between the discovery order sequence and the trapline sequence was not different than expected by chance (similarity index range, 0.29–0.67, *n* = 7 indices; *p*>0.05 for all bees, see Materials and Methods).

Third, we tested random optimization and implemented a simple random “*k*-opt” iterative improvement heuristic [12] assuming that (1) a model bee tries to improve the route between known flowers by randomly shuffling the order in which a number (*k*) of randomly selected flowers are visited and (2) the route change is kept if the new route is shorter than the previous one (otherwise it is rejected). This heuristic predicts the appearance of an optimal visitation sequence only after completion of around 100 foraging bouts, which is far higher than the 17.57±1.79 bouts (*n* = 7 bees) observed in our experiments. In general, random optimization processes do not produce stable repeatable sequences and are therefore not compatible with our data

**Figure 5:**
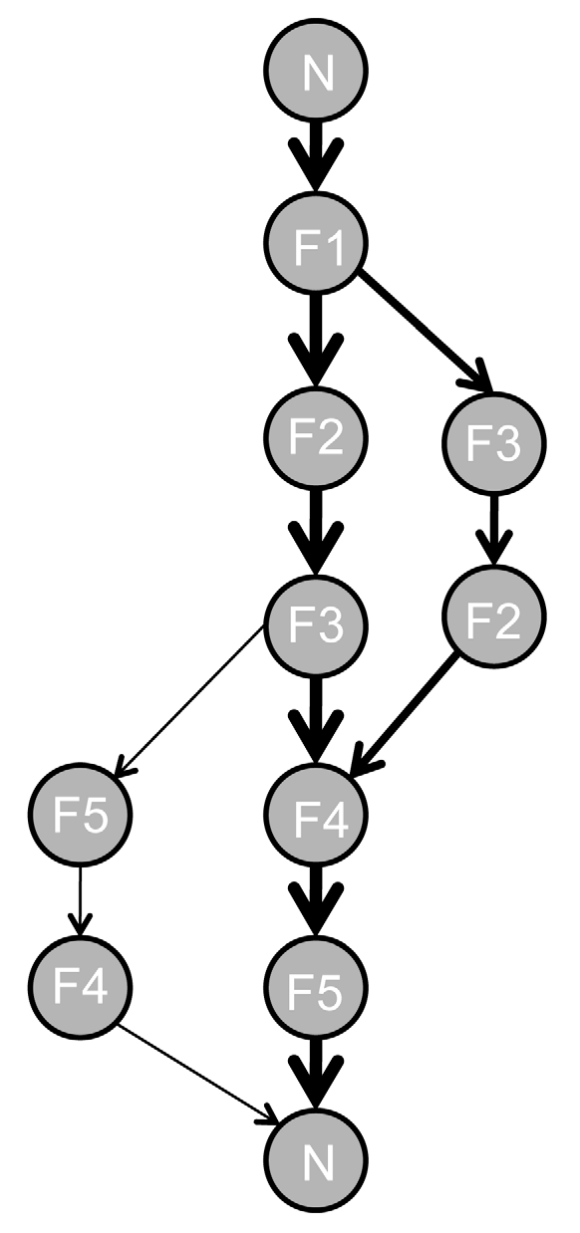
rinciple of the iterative improvement heuristic for flight path optimization. At each stage, a model bee chooses to move between flowers according to six assumptions: (1) the bee can uniquely identify each flower; (2) the bee has a finite probability of using transition vectors joining each pair of flowers; (3) the initial probability of using a vector depends on the distance between the two flowers (in our simulations nearest neighbour flowers are visited with a probability = 0.6 and more distant locations are visited with a probability = 0.1); (4) the bee computes the net length of the route travelled by summing the distances of all vectors comprising the flower visit sequence; (5) having completed a route passing through all the flowers at least once, the bee compares the net length of the current route to the net length of the shortest route experienced so far; (6) if the new route is shorter, the probabilities of using the vectors forming this new route in the next foraging bout are increased by a common factor (in our simulation, a factor of 2). The figure illustrates a late stage of trapline development between five flowers arranged in a regular pentagon (where three different paths starting and finishing at the nest-box have been selected over time (N12354N, N13245N, N12345N)). Strengthening the vectors forming the shortest route (N12345N) makes this route more “attractive” (the thickness of the arrow is proportional to the probability of the vector being used). As the bee is more likely to take the shortest route, longer routes will be gradually abandoned (see simulations in Figure S3).

We therefore developed an iterative improvement heuristic based on our analysis of bees' movement patterns. In this heuristic, the probability of model bees visiting a particular flower or flying back to the nest is determined by its experience, allowing them to explore, learn, and sequentially optimise their routes (Figure 5). We assume that (1) bees can uniquely identify the flower locations using information from path integration and/or the visual context (landmarks and/or panoramas) [26]; (2) bees have a finite transition probability between the nest and each flower and between any two flowers during the very first bout; (3) this initial probability is higher between nearest neighbour locations than between any other locations; (4) at each bout bees compute the net length of the route travelled (rather than the actual distance flown) by measuring the vector distance between successive flower visits and sum the lengths of all vectors comprising the route using path integration [27]; (5) if bees have visited all flowers at least once (and thus filled their crop), they compare the length of the current route to the memorised length of the shortest route travelled so far; and (6) if the new route is shorter, the probability of using the vectors composing this route are enhanced by a common factor.

According to our observations, a model bee during its first foraging bout between five flowers arranged in a regular pentagon is most likely to visit flowers 1 and 5 first because the other flowers are farther from the nest. Having found flower 1, the bee is most likely to find flower 2 next because flowers 3, 4, and 5 are more distant, and so on. The order in which flowers are discovered determines the probable order in which they will be visited during the next few foraging bouts; for example, from flower 1 a bee with aforementioned experience is most likely to visit flower 2 next (and more rarely move to flowers 3, 4, and 5). Nonetheless, as shown by our analyses on the flower visitation sequences of real bees (Figure 2C), these transition probabilities are not fixed and change whenever a shorter route is discovered. If a newly travelled route (e.g., sequence, 12453) is shorter than the shortest route experienced so far by that bee, then the probabilities linking movements between pairs of flowers within this circuit (1-2, 2-4, 4-5, and 5-3) are enhanced by a common factor. Gradual strengthening of the transition vectors forming the shortest route experienced so far allows the bee to sequentially optimise its visitation sequence and select an optimal route as a trapline (for more details about the model, see Text S1).

Simulation data from this novel heuristic predict that model bees (1) occasionally visit fewer than all flowers especially during early bouts, (2) regularly revisit empty flowers during the same bout, (3) decrease their frequency of revisits with experience, (4) establish stable optimal routes after about 20–25 bouts, and (5) can sequentially adjust their routes to incorporate newly discovered flowers in an optimal way (Figure S3). Quantitative evaluation of the simulated data with the optimisation performance of real bees in the experimental field showed full agreement for the number of bouts to the first appearance of an optimal sequence, the number of bouts to the stabilization of an optimal sequence into a trapline, the number of different routes experienced, the net route length travelled per bout, the number of revisits per bout, and the similarity indices between successive bouts (Table 1). Therefore, bees' optimization strategy can be captured in a simple iterative improvement routine in which an individual compares the net length of their current route to the net length of the shortest route experienced so far, and increases its probability of reusing the flight vectors comprising this new route if it is shorter.

**Table 1:** Quantitative evaluation of the iterative improvement heuristic for flight path optimization.

Optimization Index	*p*_1–7_	*p*_1_	*p*_2_	*p*_3_	*p*_4_	*p*_5_	*p*_6_	*p*_7_
Number of bouts to first appearance of an optimal sequence	0.87	0.91	0.95	0.84	0.97	0.73	0.91	0.81
Number of bouts necessary for the stabilization of a sequence	0.94	0.98	0.92	0.92	0.99	0.95	0.92	n.a.
Number of different routes experienced	0.72	0.65	0.88	0.81	0.71	0.71	0.65	0.65
Total travel distance per bout		0.4	0.61	0.51	0.35	0.38	0.24	0.46
Number of revisits per bout		0.62	0.76	0.73	0.48	0.57	0.42	0.63
Similarity index between successive foraging bouts		0.34	0.39	0.36	0.35	0.29	0.29	0.44

Comparisons between empirical (*n* = 7 bees) and simulation (*n* = 1,000 runs; for details, see Text S1) data were made for six optimization performance indices. *p*_1–7_, average value for all bees (see Materials and Methods). *p*_n_, average value for bee *n*. Labels 1–7 refer to the same individuals in all figures and tables.

## Discussion

We have recorded complete flower visitation sequences and successive flight paths of bumblebees foraging in field-scale conditions, allowing us to examine the learning processes underpinning multi-destination routing strategies of animals with known foraging history. Over multiple bouts, bees minimized their overall travel distance by flying relatively straight vectors between learnt feeding locations and visiting all flowers once in a stable optimal sequence. When the spatial configuration of flowers was modified, the bees engaged in localised search flights to find new flowers.

The observed dynamic of trapline acquisition in our large-scale setup is incompatible with random movements or with an extensive exploration of all possible routes. We also ruled out the hypothesis that bees rely on a single rule of thumb such as visiting all locations in the initial discovery order or moving between nearest neighbour locations. Although a large proportion of the bees' movements involved linking nearest neighbour flowers, especially in the first few foraging bouts, this strategy alone cannot explain our data. Rather, bees developed their traplines through trial and error by combining exploration, learning, and sequential optimisation, thus confirming hypotheses derived from previous observations in smaller enclosed environments [15],[19]–[21]. Interestingly, however, the optimisation performance of bees under field-scale conditions was much higher as all the bees tested selected an optimal route as their trapline, compared to a maximum of 75% in laboratory studies using comparable numbers of feeding locations (range, 4–10 flowers) and training durations (range, 20–80 foraging bouts per bee) [15],[19]–[21]. Presumably, bees' motivation to optimise their routes increases with spatial scale because the costs of travelling long (suboptimal) distances are greatly magnified. It is also possible that celestial cues, such as the position of the sun or polarized light patterns that are not typically available in laboratory settings but are known to be involved in navigation [28],[29], allow bees to orientate more accurately and develop routes faster in natural environments.

How, then, did the bees optimise their routes? Based on our detailed analysis of bee movement patterns, we implemented a simple iterative improvement heuristic, which, when applied to our experimental situation, matched the behaviour of real bees exceptionally well. The proposed heuristic demonstrates that stable efficient routing solutions can emerge relatively rapidly (in fewer than 20 bouts in our study) with only little computational demand. Our hypothetical model implies that a bee keeps in memory the net length of the shortest route experienced so far and compares it to that of the current route travelled. If the novel route is found to be shorter, the bee is more likely to repeat the flight vectors comprising this route. Hence, through a positive feedback loop certain flight vectors are reinforced in memory, while others are “forgotten”, allowing the bee to select and stabilize a short (if not optimal) route into a trapline. These assumptions are compatible with well-established observations that bees compute and memorise vector distances between locations using path integration [30]. For instance, bees visiting the same feeders over several bouts learn flight vectors encoding both direction and travel distance to each site, by associating specific visual scenes (such as salient landmarks or panoramas) with a motor command [26],[31].

The optimisation process we describe is analogous to the iterative improvement approach developed in “ant colony optimisation” heuristics, which has been increasingly used to explore solutions to combinatorial problems in computer sciences [32]. The rationale of these swarm intelligence heuristics is based on a model describing how ants collectively find short paths between a feeding location and their nest using chemical signals [33]. “Memory” in ant colony optimisation algorithms has no neurobiological basis but instead takes the form of pheromone trails marking established routes. The shortest route becomes more attractive due to increases in pheromone concentration as multiple ants forage simultaneously along it and continue to lay pheromone, while longer routes are abandoned because of pheromone evaporation. Of course, identification of a similar iterative optimisation principle in bees, although based on very different mechanisms (bumblebees forage individually and do not recruit using pheromone trails), does not imply that bees would equal the performance of swarm algorithms in finding solutions to complex combinatorial problems. However, iterative improvement heuristics are flexible, suggesting that bees can develop functional traplines in their natural environments, where the numbers of flowers, their spatial configuration, and reward values vary over time.

The question of how spatial information is encoded and processed in an insect brain is a matter of long-standing debate [25],[34]–[37]. Recent observations of honeybees using shortcuts between separately learnt foraging locations have been interpreted as evidence for “map-like” memory [25],[35], suggesting that bees acquire a coherent representation of the spatial connectivity between important locations in their environment (such as the nest, flowers, and prominent landmarks), allowing them to compute new vectors. Although our study was not conceived to test this hypothesis, our results indicate that the routing behaviour of bumblebees can be replicated without assuming such a map-like representation of space. The proposed heuristic suggests that bees can develop optimal routes by following multi-segment journeys composed of learnt flight routines (local vectors), each pointing towards target locations (flowers) and coupled to a visual context (landmarks and/or panoramas). Such a decentralized representation of space is akin to the “route-based” navigation of desert ants, where spatial information is thought to be processed by separate, potentially modular, guidance systems [4],[34],[37],[38]. The fact that trained bees continued to visit the familiar location from which a flower had been removed (location 3) further supports the hypothesis that foragers in our experimental situation relied heavily on learnt sensory motor routines as route-based navigation constrains the ability of individuals to rapidly adjust their routes, in contrast to map-like navigation that should allow for fast computation of entirely novel solutions [36]. Future studies should clarify whether similar learning heuristics apply to insect pollinators foraging at different spatial scales and configurations, and to other animals faced with similar routing problems (e.g., hummingbirds [9], bats [7], and primates [6],[10],[13],[14]). Ultimately, characterizing the neural-computational implementation of functional multi-destination routing solutions in small-brained animals holds considerable promise for identifying simple solutions to dynamic combinatorial problems in situations lacking central control.

## Materials and Methods

### Subjects and Study Site

Experiments were carried out in a flat, open area of mown pasture (approximately 700×300 m) on the Rothamsted estate (Hertfordshire, UK; Figure 1). Global landmarks (edges between different types of cut grass, tree lines) and local features (isolated trees) were available. The observation period (October 2010) was chosen because there were very few natural sources of pollen and nectar present during this time. The radar equipment was located on the south-east corner of the experimental field to allow maximum catchment area. The *Bombus terrestris* colony was housed in a wooden nest-box located south of the experimental field. A transparent tube with shutters was fitted at the entrance to control bee traffic. Bees were individually marked with numbered plastic tags within a day of emergence from pupae in order to monitor their complete foraging history.

### Artificial Flowers and Video Tracking

Artificial flowers (Figure S1) were made of a plastic cylinder (height 8 cm) covered with a blue horizontal landing platform (diameter 6 cm). Bees could access the flowers equally well from all angles and collect a drop of 40% (w/w) sucrose solution from a yellow plastic square (2.4 cm side) in the middle of the landing platform. Each flower was positioned on top of a truncated cone-shaped support (base diameter 30 cm, top diameter 20 cm, height 18 cm) placed on the ground. A webcam (Logitech c250, Fremont, CA) was mounted directly above the centre of each flower on an independent vertical support (height 50 cm) to capture footage of bees when they visited. Webcams were fitted with light filters (neutral density 0.6) to attenuate sunlight illumination and connected to a laptop running video motion detection software (Zone Trigger 2, Omega Unfold, Quebec, Canada). A video clip (minimum duration 5 s) was recorded each time a bee moved into the camera field of view (Video S1). Recording continued until movement stopped, thus capturing complete flower visits from when the bee landed to its departure. Feeding stations were arranged sufficiently far apart (minimum distance 50 m) such that each station would be undetectable by the bee visual system from any other. The maximum dimension of a feeding station (including the flower, webcam, and laptop) was 70 cm. Given bumblebee's failure to detect targets that subtend less than ca. 3° [39], a bee should visually detect a feeding station this size from no more than 13.4 m away. Each laptop was powered by a small petrol generator (850W, length 38 cm, width 33 cm, height 32 cm) placed 10 m from the feeding station, located outside the pentagonal flower array (Figure 1). Generators provided potential local landmarks, although due to their small size they should only have been visually detectable for bumblebees at a range of 7.3 m and were therefore less prominent to bees than feeding stations. In addition, there is solid experimental evidence that bees could not visually detect feeding stations over the distances tested, since two out of three bees failed to find the new location after a displacement (see Results).

### Harmonic Radar Tracking

The harmonic radar and transponders have been previously described [23]. The radar equipment provided coverage over a range of 700 m and an altitude of about 3 m above the ground. Transponders consisted of a 16 mm vertical dipole (mass 0.8 mg) that does not affect bees' flight behaviour [24]. Individual bees were caught on departure from the colony, the transponder was attached using double-sided foam tape over the plastic number tag and released at the nest-box entrance tube. Coordinates of the transponder-tagged bee in the experimental field were recorded every 3 s by the radar with a spatial resolution of approximately ±2–3 m [40]. When the bee returned to the nest entrance, the transponder was removed before it re-entered the nest. Wind speed and direction were measured every 10 s by a recording anemometer fixed 2 m above the ground, located 10 m west of the nest-box (Figure 1).

### Experimental Procedure

Experiments were performed between 09:00 and 18:00 on days when the sun or blue sky was visible. Bees were individually pre-trained to collect sucrose solution from the five artificial flowers arranged in a linear array (150 cm length), located 50 m north-west of the nest entrance (Figure 1). Flower rewards were refilled *ad libitum* with 10 µL. The mean volume of sucrose solution ingested by a given bee during three successive foraging bouts was used to estimate its crop capacity (range = 75–100 µl) [20]. We tested seven bees, each on a different day.

In the first phase of the experiment, bees were observed foraging on the five flowers arranged in a regular pentagon (50 m side, Figure 1), until they visited all flowers in at least five consecutive foraging bouts. This required about 7 h of observation and 28.86±2.22 foraging bouts per bee (*n* = 7 bees). Each flower contained a sucrose reward equivalent to one-fifth of the test bee's crop capacity (volume range = 15–20 µl) and was refilled after each foraging bout. All departure and arrival times at the nest-box were recorded by an experimenter. Flower visits were automatically recorded using motion-activated webcams (Video S1). Flight paths of five bees were tracked with harmonic radar towards the end of training (up to four foraging bouts per bee, including the final bout).

In the second phase of the experiment, one flower was removed from location 3 and a new one was established at location 6 (Figure 1). Three bees were observed for eight additional foraging bouts in this new spatial arrangement (all these bouts were monitored by both webcam recordings and radar tracking). The five remaining bees were not tested because of insufficient daylight to pursue the observations on the day they were trained. In total, 230 foraging bouts, 1,354 video clips, and 36 radar tracks of flight paths were analysed.

### Data Analysis

#### Video data

Evaluation of the webcam video clips provided detailed information about the behaviour of bees during their visits to flowers. For each clip, we divided the landing platform of the flower into four quadrants (90° sectors) and established in which quadrant the bee landed and left the flower (Video S1).

Time-coded video clips from all flowers allowed us to reconstruct the visitation sequence for every foraging bout of each bee. We examined how a bee's tendency to repeat visitation sequences increased with experience using a similarity index described in [20], which quantifies the similarity between pairs of flower visitation sequences. This index takes into account the length of sequences and the order of visits to flowers. The similarity index ranges between 0 (completely different sequences, e.g., 123 versus 456) and 1 (identical sequences, e.g., 12345 versus 12345). To establish whether two sequences of five flower visits were significantly more similar than expected by chance, we computed 1,000 similarity indices from 2,000 visitation sequences in which a bee visited the five locations once in a random order. Because 95% of the randomly generated similarity indices fall below a threshold of 0.67, two sequences were significantly more similar than expected by chance (at the 5% level) if the similarity index is greater than this threshold.

#### Radar data

Radar tracks gave us the flight trajectory, travel distance, and ground speed for each foraging bout. For each radar track, we calculated a mean flight vector, defined as the average of all vectors formed between each point of the flight path and the barycentre (geometric centre) of the hexagon formed by the nest-box and flower locations 1–5. A bee using the shortest possible path to visit all five flowers once would have a null mean vector. Any deviation from this optimal path is revealed by a non-null vector. Videos of all radar tracks are available (Videos S2, S3, S4, S5, S6, S7, S8, S9, S10, S11, S12, S13, S14, S15, S16, S17, S18, S19, S20, S21, S22, S23, S24, S25, S26, S27, S28, S29, S30, S31, S32, S33, S34, S35, S36, S37).

#### Quantitative analysis of simulation data

Using our heuristic for bee optimization, we predicted the distribution of values for six optimization performance indices by model bees [(1) the number of bouts to the first appearance of an optimal flower visitation sequence, (2) the number of bouts to the stabilisation of a visitation sequence (when repeated in at least three consecutive bouts and all subsequent bouts are similar), (3) the number of different routes used in total, (4) the route lengths per bout, (5) the number of revisits per bout, and (6) the similarity index] foraging on five locations arranged in a regular pentagon (Table 1). The distribution was based on 1,000 simulation runs. From these data, we calculated the probability of a real bee doing at least as well as observed given that the model is correct (null hypothesis). This probability is a *p* value, so the model can therefore be rejected if this probability is less than 0.05. For the number of bouts to the first appearance of an optimal sequence, the number of bouts to the first appearance of a stable sequence, and the number of different routes used, we calculated the average *p* value for all bees. For route lengths per bout, number of revisits per bout, and similarity indices between pairs of visitation sequences, we calculated the average *p* value for each bee using (*p*1+*p*2+…*p*n)/*n* where *p*1 is the *p* value for bout 1, *p2* is the *p* value for bout 2, and so forth.

## Supporting Information

Figure S1 Artificial flowers and video tracking system. (A) Complete feeding station including an artificial flower, a webcam, and a laptop computer. (B) Details of an artificial flower. A bee equipped with a radar transponder is collecting a drop of sucrose solution from the yellow square in the middle of the blue landing platform. The webcam above the landing platform is controlled by a motion detection software (running on the laptop) that triggers the recording of a video clip when a bee lands and feeds on the flower, thus enabling us to identify the bee, the timing of its visit, and its arrival and departure directions (Video S1).
(TIF)

Figure S2 Flight paths followed by a naïve bee in the initial pentagonal array of flowers. Black dots show the position of the bee at 3 s intervals as recorded by the radar. Red dots indicate the locations of flowers (F1–F5) and the nest-box (N). Movements of the bee (black lines) were recorded during its first four foraging bouts. Stars indicate search loops between immediate revisits to flowers. Grey arrows indicate mean wind direction (degrees) and speed (m.s^−1^). The open black arrow (bottom left of each panel) indicates north. Distances are in metres. Unlike experienced bees (Figure 3), the naïve bee did not visit the five flowers in a stable, repeatable sequence and travelled more than 1,000 m per bout.
(TIF)

Figure S3 Simulated example of trapline development using our heuristic for flight path optimization. Arrows represent the movements of a model bee between the five flowers (F1–F5) arranged in a regular pentagonal array and the nest-box (N). Numbers above each diagram indicate the foraging bouts in chronological order. An optimal (shortest) route first appears at bout 18 and is stabilized (repeated in at least three consecutive bouts and all subsequent bouts are similar) at bout 25.
(TIF)

Table S1 Complete list of visitation sequences to artificial flowers. Sequences were reconstructed from a compilation of video data recorded at each flower. For each individual bee, the visitation sequences are presented in chronological order (down a column). Numbers in the table (1–6) refer to the spatial location of each flower in the experimental field (Figure 1). For each bee, the trapline (the most common five-flower visitation sequence excluding revisits) is highlighted in bold. Optimal bouts (shortest possible sequence visiting all flowers once) are highlighted in yellow. †, sequences recorded during the second phase of the experiment when the flower was removed from location 3 and a new flower was established at location 6. *, radar-tracked sequence. Labels 1–7 refer to the same individuals in all figures and tables.
(DOC)

Text S1 Numerical simulations of our iterative improvement heuristic for flight path optimization. In this text, we describe how we selected the probability values used in the simulations.
(DOC)

Video S1 Video clip of a bee visiting flower. Top view of a bee (uniquely identified by a plastic number tag) collecting sucrose solution from the middle of the circular landing platform. Recording by the webcam above the flower was triggered by motion detection software run on a laptop (Figure S1). The bee landed and left the flower from the south-west quadrant of the landing platform (181–270°). Recording date and time are displayed in the bottom left corner of the screen.
(MP4)
*Videos of radar tracks (Video S2, S3, S4, S5, S6, S7, S8, S9, S10, S11, S12, S13, S14, S15, S16, S17, S18, S19, S20, S21, S22, S23, S24, S25, S26, S27, S28, S29, S30, S31, S32, S33, S34, S35, S36, S37). Here we present links to videos of all the radar tracks (*n* = 36) overlaid on a drawing of the experimental field. Numbered circles in videos indicate the location of flowers. The blue line indicates the bee's flight paths as recorded by the harmonic radar. Pink arrows indicate wind direction and speed. Time is accelerated ten times.*
*  Links to Videos S3, S4, S5, S6, S7, S8, S9, S10, S11, S12, S13 of the radar tracks of five experienced bees (individuals 1–5) towards the end of training in the array of five artificial flowers arranged in a regular pentagon. We also provide the radar track of a naïve bee (different from the test bees) during its first foraging bout (Video S2):*

Video S2 Naive bee on foraging bout 1.
(MP4)

Video S3 Individual 1 on foraging bout 36.
(MP4)

Video S4 Individual 1 on foraging bout 37.
(MP4)

Video S5 Individual 2 on foraging bout 24.
(MP4)

Video S6 Individual 2 on foraging bout 25.
(MP4)

Video S7 Individual 2 on foraging bout 26.
(MP4)

Video S8 Individual 3 on foraging bout 22.
(MP4)

Video S9 Individual 3 on foraging bout 23.
(MP4)

Video S10 Individual 3 on foraging bout 24.
(MP4)

Video S11 Individual 3 on foraging bout 25.
(MP4)

Video S12 Individual 4 on foraging bout 37.
(MP4)

Video S13 Individual 5 on foraging bout 28.
(MP4)
* Links to Videos S14, S15, S16, S17, S18, S19, S20, S21, S22, S23, S24, S25, S26, S27, S28, S29, S30, S31, S32, S33, S34, S35, S36, S37 of the radar tracks for three experienced bees (Individuals 1–3) after removal of a flower (from location 3) and the establishment of a more distant one (at location 6):*

Video S14 Individual 1 on foraging bout 38.
(MP4)

Video S15 Individual 1 on foraging bout 39.
(MP4)

Video S16 Individual 1 on foraging bout 40.
(MP4)

Video S17 Individual 1 on foraging bout 41.
(MP4)

Video S18 Individual 1 on foraging bout 42.
(MP4)

Video S19 Individual 1 on foraging bout 43.
(MP4)

Video S20 Individual 1 on foraging bout 44.
(MP4)

Video S21 Individual 1 on foraging bout 45.
(MP4)

Video S22 Individual 2 on foraging bout 27.
(MP4)

Video S23 Individual 2 on foraging bout 28.
(MP4)

Video S24 Individual 2 on foraging bout 29.
(MP4)

Video S25 Individual 2 on foraging bout 30.
(MP4)

Video S26 Individual 2 on foraging bout 31.
(MP4)

Video S27 Individual 2 on foraging bout 32.
(MP4)

Video S28 Individual 2 on foraging bout 33.
(MP4)

Video S29 Individual 2 on foraging bout 34.
(MP4)

Video S30 Individual 3 on foraging bout 26.
(MP4)

Video S31 Individual 3 on foraging bout 27.
(MP4)

Video S32 Individual 3 on foraging bout 28.
(MP4)

Video S33 Individual 3 on foraging bout 29.
(MP4)

Video S34 Individual 3 on foraging bout 30.
(MP4)

Video S35 Individual 3 on foraging bout 31.
(MP4)

Video S36 Individual 3 on foraging bout 32.
(MP4)

Video S37 Individual 3 on foraging bout 33.
(MP4)
