# Supplementary figures and images for: Radar Tracking and Motion-Sensitive Cameras on Flowers Reveal the Development of Pollinator Multi-Destination Routes over Large Spatial Scales
Source: PLoS Biol. 2012 Sep 20;10(9):e1001392. doi: 10.1371/journal.pbio.1001392 (PMC3462218; doi:10.1371/journal.pbio.1001392)

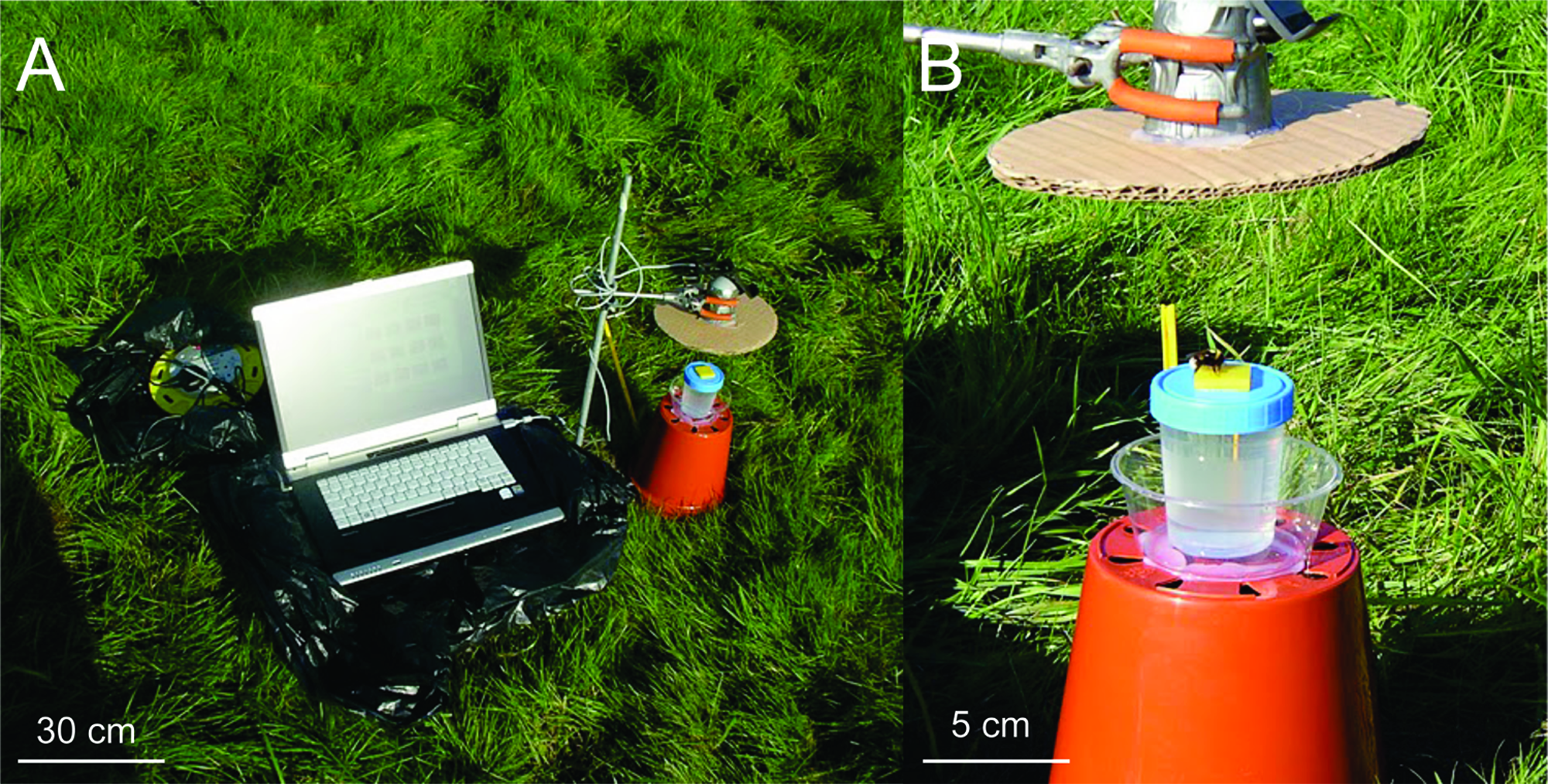

Supplement: Figure S1 — Artificial flowers and video tracking system. (A) Complete feeding station including an artificial flower, a webcam, and a laptop computer. (B) Details of an artificial flower. A bee equipped with a radar transponder is collecting a drop of sucrose solution from the yellow square in the middle of the blue landing platform. The webcam above the landing platform is controlled by a motion detection software (running on the laptop) that triggers the recording of a video clip when a bee lands and feeds on the flower, thus enabling us to identify the bee, the timing of its visit, and its arrival and departure directions (Video S1). (TIF) [file pbio.1001392.s001.tif]

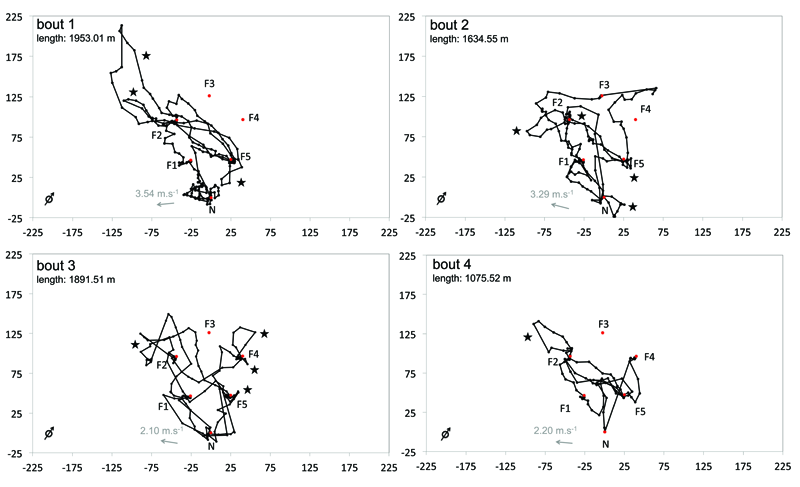

Supplement: Figure S2 — Flight paths followed by a naïve bee in the initial pentagonal array of flowers. Black dots show the position of the bee at 3 s intervals as recorded by the radar. Red dots indicate the locations of flowers (F1–F5) and the nest-box (N). Movements of the bee (black lines) were recorded during its first four foraging bouts. Stars indicate search loops between immediate revisits to flowers. Grey arrows indicate mean wind direction (degrees) and speed (m.s−1). The open black arrow (bottom left of each panel) indicates north. Distances are in metres. Unlike experienced bees (Figure 3), the naïve bee did not visit the five flowers in a stable, repeatable sequence and travelled more than 1,000 m per bout. (TIF) [file pbio.1001392.s002.tif]

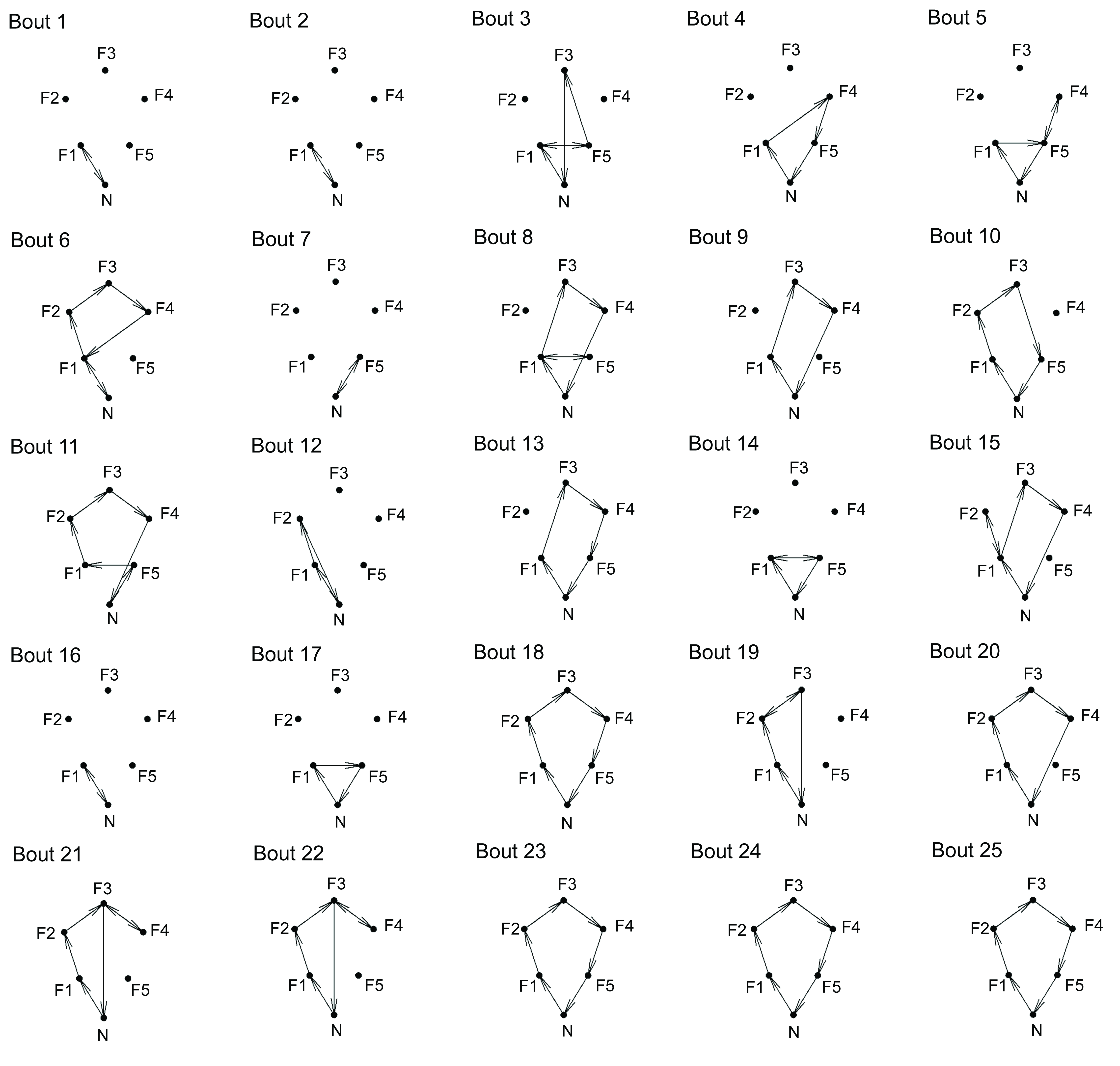

Supplement: Figure S3 — Simulated example of trapline development using our heuristic for flight path optimization. Arrows represent the movements of a model bee between the five flowers (F1–F5) arranged in a regular pentagonal array and the nest-box (N). Numbers above each diagram indicate the foraging bouts in chronological order. An optimal (shortest) route first appears at bout 18 and is stabilized (repeated in at least three consecutive bouts and all subsequent bouts are similar) at bout 25. (TIF) [file pbio.1001392.s003.tif]
