# Supplementary material for: Radar Tracking and Motion-Sensitive Cameras on Flowers Reveal the Development of Pollinator Multi-Destination Routes over Large Spatial Scales
Source: PLoS Biol. 2012 Sep 20;10(9):e1001392. doi: 10.1371/journal.pbio.1001392 (PMC3462218; doi:10.1371/journal.pbio.1001392)
Supplement: Table S1 — Complete list of visitation sequences to artificial flowers. Sequences were reconstructed from a compilation of video data recorded at each flower. For each individual bee, the visitation sequences are presented in chronological order (down a column). Numbers in the table (1–6) refer to the spatial location of each flower in the experimental field (Figure 1). For each bee, the trapline (the most common five-flower visitation sequence excluding revisits) is highlighted in bold. Optimal bouts (shortest possible sequence visiting all flowers once) are highlighted in yellow. †, sequences recorded during the second phase of the experiment when the flower was removed from location 3 and a new flower was established at location 6. *, radar-tracked sequence. Labels 1–7 refer to the same individuals in all figures and tables. (DOC) [file pbio.1001392.s004.doc]

| bout | indiv 1 | indiv 2 | indiv 3 | indiv 4 | indiv 5 | indiv 6 | indiv 7 |  | naïve |
| --- | --- | --- | --- | --- | --- | --- | --- | --- | --- |
| 1 | 15 | 5 | 15121555335115515 | 15154 | 1 | 155 | 51 |  | 5522522521 * |
| 2 | 111 | 1 | 1141 | 51544515514445 | 111554 | 1154114 | 1511111515 |  | 5522253121 * |
| 3 | 111 | 1 | 5 | 551224 | 133 | 5152 | 111112221 |  | 12215544425 * |
| 4 | 1511 | 512 | 2355433523455433 | 5415451 | 113 | 233235 | 13331 |  | 52252154 * |
| 5 | 525511 | 55551121 | 55 | 54415 | 122 | 12 | 12215 |  |  |
| 6 | 54332 | 52 | 335 | 515454 | 1221221 | 425132 | 155512334 |  |  |
| 7 | 115332 | 55 | 543 | 5441 | 5122 | 523455324512 | 1235 |  |  |
| 8 | 144321 | 514 | 5433 | 1541545415445415 | 12222344 | 432421554325 | 1245 |  |  |
| 9 | 1332 | 5122 | 4355 | 51 | 123331244123 | 412 | 1245511 |  |  |
| 10 | 133215421 | 21 | 5433 | 1414 | 122233221 | 52453241 | 123 |  |  |
| 11 | 133254 | 5221 | 4543 | 121 | **12223345** | 5233 | 1235 |  |  |
| 12 | 134 | 52221 | 4452 | 412 | **123345**123 | 25224 | 1245 |  |  |
| 13 | 1234432 | 521 | 5442 | 441 | 1232354 | 145125 | **12345**1 |  |  |
| 14 | 11233223445 | 512 | 543 | 14414121154255255123 | **123445** | 12321245 | **123445** |  |  |
| 15 | 14511 | 5125 | **54321** | 5431533521532254324 | 125543 | 1234423451 | **1234512345** |  |  |
| 16 | 1325 | 521 | 5421 | 555432 | **123445**51 | 1434515134515 | **12345** |  |  |
| 17 | 135 | 521 | **54321**42 | 123355 | 2345 | 11 | 12354 |  |  |
| 18 | 1351134 | 52 | **54321** | 12233325 | **1123445** | 542 | **12345** |  |  |
| 19 | **12345** | 522152215 | 32 | 5432 | 124512 | **12345** | **12345** |  |  |
| 20 | 54321 | 52232255 | **543321** | 543235 | 1245 | 12511212445112 | 1345 |  |  |
| 21 | 14321 | 52354521 | **54321** | 5122351232543 | 13215 | 3451 | 1234**12345** |  |  |
| 22 | 1432 | 5431251324334 | 4321 * | 543235 | **12345** | **123451234512345** | **123512345** |  |  |
| 23 | 13432 | **54321** | **54321** * | 54325**54321**421 | 12551353223 | **12345** |  |  |  |
| 24 | 13 | **54321** * | **54321** * | 5122325332533554 | 12245512351 | **1234512345** |  |  |  |
| 25 | 134321 | **543211** * | **54321** * | 5433 | **12345**121 | **12345** |  |  |  |
| 26 | 1234 | **54321** * | 542154442154 † * | 543 | **123344512345** | **12345** |  |  |  |
| 27 | 13223414321 | 5421 † * | 542 † * | 54331 | **1234512345**1 | **12345** |  |  |  |
| 28 | 1233 | 5422115421542221 † * | 55421 † * | **54321** | **12345** * |  |  |  |  |
| 29 | 1341 | 542154221 † * | 544 † * | **54321** |  |  |  |  |  |
| 30 | 2345 | 542154215421542211 † * | 542544 † * | 543251 |  |  |  |  |  |
| 31 | 12341 | 55211 † * | 54 † * | 54323 |  |  |  |  |  |
| 32 | 124345 | 542115421 † * | 5421544254 † * | **54321** |  |  |  |  |  |
| 33 | **12345**4 | 542221 † * | 5421 † * | **54321** |  |  |  |  |  |
| 34 | **12345** | 5422154 † * |  | **54321** |  |  |  |  |  |
| 35 | **12345** |  |  | **54321** |  |  |  |  |  |
| 36 | **123345** * |  |  | **54321** |  |  |  |  |  |
| 37 | **12345** * |  |  | **54321** * |  |  |  |  |  |
| 38 | 1244512466 † * |  |  |  |  |  |  |  |  |
| 39 | 124565 † * |  |  |  |  |  |  |  |  |
| 40 | 1244665 † * |  |  |  |  |  |  |  |  |
| 41 | 124665 † * |  |  |  |  |  |  |  |  |
| 42 | 12466421 † * |  |  |  |  |  |  |  |  |
| 43 | 12465 † * |  |  |  |  |  |  |  |  |
| 44 | 124651 † * |  |  |  |  |  |  |  |  |
| 45 | 12465 † * |  |  |  |  |  |  |  |  |
