# Supplementary material for: Radar Tracking and Motion-Sensitive Cameras on Flowers Reveal the Development of Pollinator Multi-Destination Routes over Large Spatial Scales
Source: PLoS Biol. 2012 Sep 20;10(9):e1001392. doi: 10.1371/journal.pbio.1001392 (PMC3462218; doi:10.1371/journal.pbio.1001392)
Supplement: Text S1 — Numerical simulations of our iterative improvement heuristic for flight path optimization. In this text, we describe how we selected the probability values used in the simulations. (DOC) [file pbio.1001392.s005.doc]

**Text S1 Numerical simulations of our iterative improvement heuristic for flight path optimization.** In the simulations a bee initially moves from the nest to flower 1 with probability of 0.8, and from the nest to flower 5 with probability of 0.2, because flower 1 was found before flower 5 and subsequently becomes a preferred first port of call. Whether flower 1 or flower 5 is chosen as the first port of call does not affect model predictions for performance in optimising sequences. During each subsequent move the bee flies to the nearest flower (or the nest if this is nearest) with probability 0.6, and to any of the more distant flowers (or nest if more distant) with probability 0.1. A foraging bout ends if the bee returns to the nest or if it has visited all five flowers (and so filled its crop). Transition probabilities are enhanced by a factor of 2 if the route length is less than that of the shortest route found so far (provided all five flowers were visited). All transition probabilities are then re-scaled so that they sum to 1 at the nest and at each flower.

Numerical simulations are in good agreement with our observations of real bees (Table 1). Note, however, that our observational data for the numbers of bouts taken to establish a stable trapline are only sufficient to demonstrate that the model is in good agreement with average bee behaviour. This is because our observational data for this trait does not capture atypical, far-from-average behaviour. The observed numbers of bouts (20 to 33) does not extend far beyond the 95% confidence interval (22-32) for the mean number of bouts. Nonetheless, our observations do capture some far-from-average behaviour and so for these traits are sufficient to properly test the model. The range of the observed number of bouts before the first appearance of an optimal trapline (14-28) does, for example, fall outside of the 95% confidence interval for the mean number of bouts (16-20). The good model agreement with these observational data demonstrates that the model captures both typical and atypical behaviour. The model predictions are robust to variation in the choice of initial values for the transition probabilities and/or the probability enhancement factor. Comparable agreement between model predictions and observations (as quantified by *p*-values) are obtained for probability enhancement factors in the range 1.5 to 4 and for initial transition probabilities to the nearest flowers in the range 0.55 to 0.65.
